# Assessment of GeneXpert GxAlert platform for multi-drug resistant tuberculosis diagnosis and patients’ linkage to care in Tanzania

**DOI:** 10.1186/s13104-018-3235-7

**Published:** 2018-02-09

**Authors:** Nicholaus Peter Mnyambwa, Issack Lekule, Esther S. Ngadaya, Godfather Kimaro, Pammla Petrucka, Dong-Jin Kim, Johnson Lymo, Rudovick Kazwala, Fausta Mosha, Sayoki G. Mfinanga

**Affiliations:** 1 School of Life Sciences and Bioengineering, Nelson Mandela African Institution of Science and Technology, Arusha, Tanzania; 2 National Institute for Medical Research, Muhimbili Medical Research Centre, Dar es Salaam, Tanzania; 3 Kibong’oto Infectious Diseases Hospital, Kilimanjaro, Tanzania; 4 College of Nursing, University of Saskatchewan, Saskatoon, Canada; 5 National Tuberculosis and Leprosy Programme, Dar es Salaam, Tanzania; 6 Faculty of Veterinary Medicine, Sokoine University of Agriculture, Morogoro, Tanzania; 7 National Health Laboratory, Ministry of Health Community Development Gender Elderly and Children, Dar es Salaam, Tanzania

**Keywords:** Multi-drug resistant tuberculosis (MDR-TB), MDR-TB treatment, Tuberculosis (TB) diagnosis, GeneXpert, GxAlert, Rifampicin resistant

## Abstract

**Objective:**

The gap between patients diagnosed with multi-drug resistant tuberculosis (MDR-TB) and enrolment in treatment is one of the major challenges in tuberculosis control programmes. A 4-year (2013–2016) retrospective review of patients’ clinical data and subsequent in-depth interviews with health providers were conducted to assess the effectiveness of the GeneXpert GxAlert platform for MDR-TB diagnosis and its impact on linkage of patients to care in Tanzania.

**Results:**

A total of 782 new rifampicin resistant cases were notified, but only 242 (32.3%) were placed in an MDR-TB regimens. The remaining 540 (67.07%) patients were not on treatment, of which 103 patients had complete records on the GxAlert database. Of the 103 patients: 39 were judged as untraceable; 27 died before treatment; 12 were treated with first-line anti-TBs; 9 repeat tests did not show rifampicin resistance; 15 were not on treatment due to communication breakdown, and 1 patient was transferred outside the country. In-depth interviews with health providers suggested that the pre-treatment loss for the MDR-TB patients was primarily attributed to health system and patients themselves. We recommend strengthening the health system by developing and implementing well-defined interventions to ensure all diagnosed MDR-TB patients are accurately reported and timely linked to treatment.

## Introduction

Tuberculosis (TB) remains an important health problem worldwide. Effective TB control interventions require early identification of TB infected persons and tailored therapies. As a result of limited performance of smear microscopy [1, 2] and a slow generation time of mycobacteria on culture media [3], the WHO endorsed the use of GeneXpert MTB/RIF for rapid detection of TB and rifampicin resistance [4]. As rapid diagnostic tools become available, the main challenge remains on how responsive the health system is to ensure effective linkage of diagnosed patients to requisite healthcare.

Tanzania is ranked among 20 countries with the highest TB incidences worldwide [5]. An increase in MDR-TB cases within the country was first noted in 2005, resulting in the concerted efforts by the Ministry of Health, which include designation of Kibong’oto Infectious Diseases Hospital (KIDH) as the first centre for treatment of MDR-TB [6]. In 2013, a pilot GxAlert health platform was installed connecting GeneXpert diagnostic devices with the aim of monitoring GeneXpert results and improving the linkage of TB patients to care as facilitated by the real-time reporting of diagnosis results hence timely decision making. The GxAlert initiative is an electronic system for data management, designed to enable a range of TB, HIV, and Ebola diagnostic devices to connect to the network/mobile channels [7]. Results from the GeneXpert device is automatically sent to the GxAlert, in which a short message is generated and sent through mobile channels or over internet as e-mail to referring clinicians, treatment centres, and the country’s existing health information systems. Ideally, this systematic approach would contribute to the patients being diagnosed and managed in a timely and consistent manner. This study was conducted to assess the effectiveness of GeneXpert GxAlert health platform for MDR-TB diagnosis and its facilitation of the linkage of patients to healthcare services.

## Main text

### Materials and methods

#### Design

This is a retrospective review of routine clinical data of a cohort of MDR-TB patients diagnostically confirmed by the GeneXpert and complemented with interviews with health providers: Regional TB Leprosy Coordinators (RTLCs) and District TB and Leprosy Coordinators (DTLCs) from all parts of the country where patients were not enrolled in treatment. TB services are provided free of charge and coordinated by DTLCs and RTLC coordinators at district and regional levels, respectively. Additionally, efforts were made to reach the patients through by phones and/or physically whenever necessary.

#### Study population

A cohort of MDR-TB patients with a confirmed GeneXpert diagnosis between January 01, 2013 and September 21, 2016.

#### Data collection

A list of rifampicin-resistant patients with complete information from the GxAlert system was formulated from the CTRL MDR-TB database, which is composed of test results from all networked GeneXpert within Tanzania. Patients’ information was extracted from TB treatment (KIDH) and laboratory registries as well as from the GeneXpert machines. Information extracted included patient demographic information (age, sex, and contact/physical address), place of diagnosis, diagnosis results and whether the patient was placed in treatment or not.

#### Data management

Before analysis, all data from relevant hospital records were cleaned by checking for accuracy, and completeness. Recorded audio of in-depth interviews (30–45 min) with key informants were translated from Swahili to English. Thematic analysis was conducted for themes, patterns, and organized into main thematic categories.

#### Key informants knowledge of MDR-TB diagnosis and management

Assessment of level of knowledge of the MDR-TB was based on diagnosis and management of MDR-TB. A good knowledge included whether the coordinator was able to interpret GeneXpert results, aware that sputum sample of every TB patient should be sent to CTRL for culture, and Drug Sensitivity Testing, and arranging placing transport order from KIDH to pick an MDR-TB patient up to KIDH for treatment initiation.

### Results

**Fig. 1:**
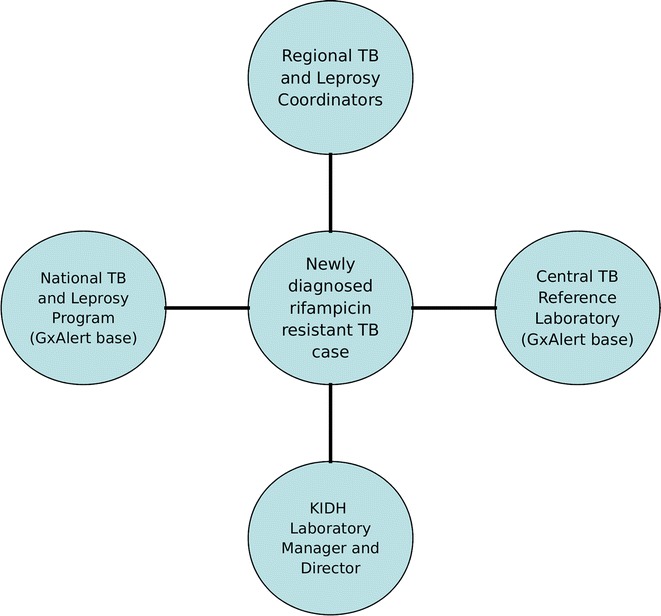
The visualization of the TB ‘spill out’ of the GxAlert notification process in Tanzania

By the end of 2016, there were 70 GeneXpert devices, of which 37 were connected with GxAlert. Figure 1 summarizes the MDR-TB notification process to clinicians and TB program officials in Tanzania. A monthly summary of diagnosis results was produced from each diagnostic centre with GeneXpert not connected to GxAlert. These results were aggregated into the GxAlert database using Xpert tracking tool. A total of 878 rifampicin resistant cases were registered in the GxAlert database between January 01, 2013 and September 21, 2016. Out of the 878 patients, 58 were identified as duplicates and 38 samples for External Quality Assurance processes. The remaining 782 were verified unique patients registered in the GxAlert; however, only 242 (32.3%) were found to be in MDR-TB treatment regimen at KIDH, leaving 540 (67.7%) patients outside the requisite continuum of care. Amongst the 540 patients, only 103 had all required information (including patients’ name and identification) at CTRL. The remaining 437 were considered incomplete records; of which twenty-five (25) were missing both names and identification.

Of the 103 patients with complete records, 27 died before MDR-TB treatment initiation with 69 days mean duration from the diagnosis date; 39 patients had no medical records available, and 12 patients were treated with first-line anti-TB drugs. Of the 12 patients treated with first-line anti-TB drugs, 5 had repeat test rifampicin resistance not detected; 4 had no records available; 2 results were not correctly recorded in the laboratory register; and 1 case was confirmed MDR-TB, but the DOT nurse did not interpret the result correctly, and the patient was put on first-line anti-TB drugs. Nine patients were not placed on MDR-TB treatment because GeneXpert repeat tests showed no resistance. Of all 103 patients, 11 patients were successful traced by the study team and were not in treatment due to communication breakdown among health providers (laboratory technicians, DOT nurses, DTLCs) and patients. These 11 patients were subsequently referred to their respective DTLC for treatment procedure. Another 4 patients were not in treatment because results were not communicated to DTLCs. One patient from Morogoro was initiated with first-line anti-TB agents and later transferred by family to South Africa for treatment (Table 1).

**Table 1:** Distribution of patients with complete records

Region	Died	Not in treatment due to miscommunication	Not traceable	Placed on 1st line anti-TBs	Repeat tests rifampicin resistant not detected	Total
Tabora	0	0	0	1	0	2
Shinyanga	2	1	1	0	2	8
Simiyu	1	0	0	0	0	1
Mwanza	2	0	0	0	0	2
Geita	0	0	2	1	0	4
Kagera	1	0	0	1	0	2
Songea	0	1	1	1	1	4
Mbeya	3	0	0	0	5	8
Katavi	1	0	1	0	1	3
Kilimanjaro	0	0	0	0	0	4
Tanga	1	1	0	0	0	2
Manyara	0	0	0	2	0	2
Pwani	0	0	0	1	0	1
Lindi	0	0	1	1	0	2
Mtwara	1	1	0	1	0	3
Morogoro	3	2	0	0	0	5
Dar es Salaam	12	9	33	3	0	58
Total	27	15	39	12	9	102

Medical records for 39 patients were missing and patients were not reachable through mobile phones and physical addresses. Of the 103 patients, only 11 were interviewed by the research team as a complement to factors associated with pre-treatment loss

#### Key informants (KIs)

Twenty-seven (27) TB coordinators at the district and regional levels were interviewed to gather information on their knowledge of MDR-TB management and challenges for effective program implementation. Out of these 21 (77.8%) were males with mean age of 47.5 ± 8.8 and average of 7.7 ± 6.1 years work experience as TB coordinators. Of all TB coordinators, 17 (63.0%) were DTLCs while 10 (39.0%) were RTLCs. At the time of data collection, only RTLCs were connected to the GxAlert notification system: all acknowledged receiving short text notifications via mobile channels when an MDR-TB case was detected by GeneXpert, and communicated with respective DTLC and KIDH. All had good knowledge of MDR-TB patient management, while most (70.4%, n = 20) were aware of the classification of GeneXpert results and treatment regimens.

Following an in-depth interview with key informants and review of patients’ records, factors associated with pre-treatment loss were either related with the health-system, patient, social issues, or death of patient before taken to KIDH. Inadequate patient’s records, miscommunication between health providers, failure to assess “low” and “high” likelihood of MDR-TB and lack of transportation to KIDH were noted during interviews.

One KI said ‘Results obtained on September 28, 2015, and the patient died on November 2, 2015, before taken to KIDH… another MDR patient who was an antiretroviral therapy defaulter, he did not start the treatment as he was not reported to the DTLC after the results’.

Some MDR-TB patients were reluctant to provide correct contact addresses fearing disclosure of their health condition to relatives. In some cases, the illness is attributed to superstition.

‘…this patient was a traditional healer, after a couple of days, his condition became worse; he decided to contact RTLC for help. RTLC made a quick logistical arrangement for a car from KIDH, but the patient died before taken to KIDH’. KI said.

By the end of 2015, there was only one hospital mandated to initiate MDR-TB treatment, so social issues (e.g., parenting) related to separation for prolonged MDR-TB treatment and hospitalization away patient’s residence was highly complicated. For example, some worried losing their jobs.

‘You know some patients are heads of households and children depend on them, they refuse to leave their families to KIDH for a long duration of treatment’. KI said.

## Discussion

The findings of this study demonstrate that the installation of the GxAlert and GeneXpert enhance rapid diagnosis of TB and improve communication of the results among health providers. This linkage could ideally improve patient’s follow-up and timely access to healthcare. However, of the MDR-TB patients diagnosed by the GeneXpert between January 2013 and September 2016, only one-third were placed into care. This reflect a small proportion of a total number of MDR-TB patients diagnosed during the same time period in the country. Similarly, in 2015 the WHO reported only 125,000 (20%) of 580,000 new patients eligible for MDR-TB treatment globally were placed on treatment [5]. This persistent and complex gap perpetuates negative implications on the transmission, severity, and prognosis as well as an ethical dilemma since any diagnosed patient deserves to be provided with appropriate treatment.

Despite the added advantage of access to real-time information through GeneXpert GxAlert platform, obstacles such as inaccurate or incomplete patient data entry during the operation of GeneXpert “garbage in, garbage out”, and communication breakdown among health providers (laboratory technicians, referring clinicians, TB coordinators), hinder an effective program implementation. More than two-thirds of patients had incomplete information in the GxAlert database. This scenario contributes to a delay, pre-treatment loss and/or non-standardized therapy, which, in turn, may contribute to continued transmission, development of drug resistance and high mortality rates among MDR-TB patients [8]. Treating MDR–TB patients with first-line anti-TBs may worsen patient’s health, make the disease difficult to cure and death. Limited knowledge among laboratory personnel on the new TB coding systems resulted in misreporting and inconsistency in reporting the GeneXpert results. Misreporting of patients refrained some of the patients from getting proper health care. Successful MDR-TB treatment requires adherence to the MDR-TB guideline such as early detection and referring an MDR-TB patient to a specialized TB centre for treatment initiation with second-line anti-TB drugs, with close monitoring of the treatment outcome [9]. As the Ministry of Health works to scale up diagnostic platform, harmonization of the recording system, inclusion of DTLCs in the GxAlert system, and training to all DOT nurses, TB coordinators, and laboratory technicians on the new diagnostic algorithm and management of TB/MDR-TB could significantly improve provision of healthcare services.

Traditional beliefs and superstitions, as well as limited knowledge on transmission and treatment of the disease, stigma related to TB/MDR-TB, and opting alternative treatment, might have contributed to pre-treatment loss. Healthcare seeking behaviors among TB patients correlate with their level of knowledge and awareness of TB [10]. Stigmatization of MDR-TB patients, due to fear of spread of infection, has been elsewhere reported as contributing to treatment delay [11, 12].

For the period 2013–2015, KIDH was the only specialized reference hospital for the treatment of MDR-TB. The ongoing effort to decentralize MDR-TB therapy should aim at expanding services to the district level, hence providing choice for MDR-TB patients where to get treatment and making ‘nearer’ to home services a reality. The duration for MDR-TB treatment takes at least 20 months [13], and a patient remain hospitalized until the sputum smear become negative, which takes more than 2 months [14].

Our findings revealed that MDR-TB patients often spend over 2 months with their families and die before getting appropriate treatment. If these patients were to receive treatment in timely manner, most of these deaths could have been averted. The delays can be attributed to both patients, and the health system [15].

### Conclusion

The study revealed a significant proportion of MDR-TB patients were not enrolled in treatment often attributable to; substantial inconsistencies and deficiencies in the reporting of diagnosis results from the GeneXpert. Training of laboratory technicians and clinicians involved in the TB program and strengthening the health community workers for TB patients tracing is recommended.

## Limitation

Patients untraceability prevented interviews that could preciously  establish factors associated with pre-treatment loss; hence there is potential bias. Diagnosis rate and timing, as well as treatment outcomes, were not assessed.
